# Students’ Perceptions of Effective Math Learning Strategies

**DOI:** 10.3390/bs15081047

**Published:** 2025-08-01

**Authors:** Marissa K. Hartwig, Doug Rohrer

**Affiliations:** 1 Department of Psychology, University of Maryland, College Park, MD 20742, USA; 2 Department of Psychology, University of South Florida, Tampa, FL 33620, USA; drohrer@usf.edu

**Keywords:** math learning, spaced practice, interleaved practice, student perceptions, metacognition

## Abstract

Two highly effective math learning strategies are *spaced practice* (in which problems of the same kind are distributed across many sessions) and *interleaved practice* (in which problems of different kinds are mixed rather than blocked). Though these strategies are supported by data, students may be reluctant to use them if they perceive the strategies as ineffective or unpleasant. In Study 1, we surveyed 174 grade 7 math students about the efficacy and likability of spaced and interleaved practice. Spaced practice was often judged likable, but nearly half of students failed to recognize its efficacy. Interleaved practice was judged both unlikable and inefficacious by most students. In Study 2, we further explored perceptions of interleaving in a survey of 233 grade 7 math students. Again, students erroneously judged interleaved practice to have low efficacy. Compared to blocked practice, interleaved practice was judged less effective, less preferable, more time-consuming, and more difficult. This work identifies perceptions that may discourage students from using effective learning strategies and also shows that specific perceptions differ by strategy. Helping students overcome their negative perceptions of spacing and interleaving is an important future direction.

## 1. Introduction

Considerable research has identified learning strategies that are reliably effective across various learning conditions, tasks, and materials (for a review, see Dunlosky et al., 2013), but much less is known about students’ perceptions of these strategies, which can affect whether students use them. Two highly effective strategies for math learning are *spaced practice* (in which problems of the same kind are distributed across multiple sessions) and *interleaved practice* (in which problems of different kinds are mixed rather than blocked). The evidence for these two strategies is strong, as reviewed below, but empirical efficacy and expert recommendation is not sufficient to ensure utilization. Students may be reluctant to use strategies they perceive as ineffective, difficult, or unpleasant, and even teachers may be disinclined to impose strategies they know their students dislike. Thus, in two studies presented here, we examined students’ perceptions of spaced and interleaved math practice.

### 1.1. Evidence for Spaced and Interleaved Practice

Both spaced practice and interleaved practice are supported by a wealth of empirical evidence and have been widely recommended for use in classrooms (e.g., Carpenter, 2014; Carpenter et al., 2012; Kang, 2016; Roediger & Pyc, 2012; Rohrer, 2012, 2015; Weinstein et al., 2018). Here we provide only a brief overview of the evidence, as numerous authors have published reviews pertaining to the efficacy of spaced and interleaved^1^ practice (e.g., E. L. Bjork & Bjork, 2011; Carpenter et al., 2012, 2022; Cepeda et al., 2006; Dunlosky et al., 2013; Firth et al., 2021; Kang, 2016; Roediger & Pyc, 2012; Son & Simon, 2012). Though these strategies can support learning in many different content areas, most important for the present research is that these strategies have been demonstrated to be highly effective for math learning, as we describe next.

In math spacing experiments, practice problems of one kind (e.g., adding fractions) are either distributed more widely (e.g., across 2 weeks) or concentrated into a shorter period (e.g., across 2 days), and results typically show that final test scores are better when practice is more distributed (spaced), despite equating the number of practice problems and test delay. The efficacy of spaced practice for math problem solving has been demonstrated in laboratory experiments (e.g., Gay, 1973; Rohrer & Taylor, 2006, 2007) and classroom studies (e.g., Barzagar Nazari & Ebersbach, 2019; Emeny et al., 2021) with students ranging from grade schoolers (Schutte et al., 2015) to college students (Hopkins et al., 2016; Lyle et al., 2020) and with a variety of math topics (e.g., geometry, algebra, diagrams, permutations, pre-calculus; see Carpenter et al., 2022). According to various theories of the spacing effect (for a review, see Delaney et al., 2010), spaced practice gives students a mental break that permits renewed attention in subsequent sessions and memory consolidation in the interim, as well as promoting varied memory cues in different learning contexts and retrieval of information from earlier sessions—all of which can support learning and retention of studied materials. No single explanatory theory of spacing is agreed upon, and multiple mechanisms may operate concurrently, but the benefit of spacing is undisputed. To achieve durable learning, math assignments should be spaced such that learners must revisit and reapply math concepts and strategies across time.

When math practice is interleaved, students practice a mix of different problem types within the same assignment (e.g., adding fractions, solving proportions, and computing slope), compared to assignments consisting of blocks of one type (e.g., all slope problems). Interleaved math practice has been shown to reliably produce better test scores than blocked practice in laboratory experiments (e.g., Mayfield & Chase, 2002; Rohrer & Taylor, 2007) and classroom studies (e.g., Rohrer et al., 2015, 2020b) with students of different ages (e.g., Foster et al., 2019; Taylor & Rohrer, 2010) with topics of varying complexity and similarity (e.g., Rohrer et al., 2014; Sana et al., 2017) and at varying test delays (e.g., Rau et al., 2013; Ziegler & Stern, 2014). Interleaved math practice helps students learn to identify appropriate strategies for each problem, rather than merely repeating the procedure of the preceding problem; it also inherently increases the spacing of each problem type and encourages students to try to retrieve the needed information (e.g., fact, formula, procedure) from memory (see Rohrer et al., 2020b, for further discussion). In short, interleaved math practice entails a combination of strategy selection, spacing, and retrieval that yields sizable benefits for students’ test performance.

Spaced and interleaved math practice are strongly supported by evidence and recommended for use in math classrooms and textbooks (Carpenter, 2014; Dunlosky, 2013; Pan, 2015; Roediger & Pyc, 2012; Rohrer et al., 2020a), but recommendations alone may not be enough. Whether strategies get used in classrooms can, in part, be determined by the experiences that students and teachers have with those strategies. Do the strategies feel effective? How likable are they? If a strategy seems ineffective or unpleasant for students, then students (or their teachers) might choose other—possibly less effective—strategies that students find preferable.

### 1.2. Perceptions of Spaced and Interleaved Practice

Student perceptions of learning strategies influence which strategies they use. Models of self-regulated learning predict that student metacognitive knowledge or beliefs about learning activities affect study strategy decisions and motivation (e.g., Zimmerman, 2000; for a review of models, see Panadero, 2017). Unfortunately, explicit instruction about effective learning strategies is rare in classrooms, instead leaving students to rely on prior experience, intuitions, and heuristics—all of which can produce flawed inferences. Students’ experiences with learning strategies do not typically occur under isolated and controlled conditions that would permit accurate comparisons of efficacy. Students’ attempts to judge strategy efficacy may be further thwarted when learning outcomes are not immediate or if the student has previously succeeded despite using poor strategies. How a strategy feels while using it can also lead students astray: when effective study strategies are perceived to require more effort, students may perceive them to be less efficacious or efficient (R. A. Bjork et al., 2013; Rea et al., 2022). Students may rely on other shortcuts as well to judge strategy efficacy (e.g., how their peers study), which can yield poor guidance. In a framework for improving student self-regulation of learning strategies, McDaniel and Einstein (2020) proposed conditions needed to support students’ use of effective strategies, including the belief that a strategy is effective, knowledge about how to use it, commitment to using it, and a plan to implement it. Undoubtedly, students’ perceptions of strategy efficacy are fundamental to fostering the use of effective strategies.

Do students perceive spacing and interleaving to be efficacious for learning? Surveys of college students’ study behaviors and beliefs provide insight. With respect to spacing, 85% of college students said spaced study was better than massed study for long-term retention of material (Susser & McCabe, 2013), and 81% said flashcards should be spaced rather than massed (Wissman et al., 2012). When asked to judge a vignette comparing spaced and massed practice, 69% of college students judged spaced practice to be more effective (Morehead et al., 2016). This evidence suggests that students have some awareness of the efficacy of spaced practice. However, though many students may correctly identify spaced practice as more effective than massed practice, they may not recognize the benefit of practice that is more spaced versus less spaced. For instance, when asked to depict ideal spacing or to hypothetically schedule study activities to maximize exam performance (e.g., Blasiman et al., 2017; Cohen et al., 2013; Hartwig et al., 2022; Taraban et al., 1999; Wissman et al., 2012), many college students opted for study to be concentrated (i.e., less spaced) near exams rather than spacing those same study activities across a longer time period.

With respect to interleaving, perceptions of efficacy may be especially lacking. In lab studies, participants have been asked to judge interleaving efficacy after using both interleaved and blocked practice to learn to classify novel paintings by artist style (e.g., Kornell & Bjork, 2008; Kornell et al., 2010; Yan et al., 2016). In these studies, many participants incorrectly believed that blocked practice had been more effective than interleaved—even though their own test performance showed the opposite—and were not easily convinced of interleaving’s superiority (Yan et al., 2016). In other studies, when given a vignette describing interleaved and blocked practice for the artist learning task, only 16% of college students correctly judged interleaved practice to be more effective (Morehead et al., 2016; see also Halamish, 2018; McCabe, 2011). The perceived superiority of blocked practice is not limited to the artist learning paradigm. Studies involving hypothetical scheduling of exam preparation have also shown that participants commonly select blocked over interleaved practice (Hartwig et al., 2022; Yan et al., 2017; Yan & Sana, 2021).

Even in a familiar subject area like mathematics, in which K-12 and college curricula commonly involve revisiting concepts and skills across time (spacing) and reviewing a mix of problem types before exams (interleaving), research suggests that students still undervalue these strategies. In one recent study (Hartwig et al., 2022), college students were asked to create or select hypothetical 2-week schedules that they believed would maximize performance on a final math test. The authors then computed the degree of spaced and interleaved practice in those schedules and found substantially less spacing and interleaving than would be optimal for learning. To the degree that participants’ schedules provided spacing or interleaving, it was mostly due to the inclusion of a pre-exam review session intended to refresh one’s memory on each problem type shortly before the test, rather than a sophisticated understanding of the efficacy of spacing practice across time or interleaving problem types within sessions. In short, spaced and interleaved practice are mistakenly judged to have low efficacy in many contexts, including math learning.

Student enjoyment of learning strategies may also be an important perception that can affect their strategy choices, though this has not been the subject of much previous investigation. There is some evidence that students choose learning strategies they find more enjoyable (e.g., Obergriesser & Stoeger, 2020). Further, it is plausible that the expected enjoyment (or perceived likability) of a learning strategy may be related to other perceptions—such as the perceived efficacy or perceived difficulty of the strategy. How (or whether) such perceptions are related to one another and shape students’ preferences for spaced and interleaved math practice remains largely unexplored—but could provide insights about students’ willingness to use these highly effective strategies.

Of course, the role of students in deciding which strategies they use can differ by situation and change over time, with students typically exercising more control as they advance in school and eventually, as young adults, making many study decisions on their own. In the present studies, we sampled students in middle school—an age at which students may develop learning habits and strategy perceptions that persist into high school and college. Knowing middle schoolers’ perceptions might allow early targeting of interventions, possibly improving academic outcomes over a longer trajectory. Certainly, teachers play an important role in shaping students’ strategy use, if not through direct instruction about learning strategies, then via the lessons, assignments, and other work or activities they plan. Though such plans are teacher-generated, teachers are not impervious to students’ opinions and may plan tasks they believe their students will perceive positively. Students’ perceptions of these tasks can affect their engagement in class and also which strategies they adopt when studying on their own. Whether by teachers’ decisions or students’ own decisions, student perceptions of learning strategies can influence which strategies get used.

### 1.3. Overview of the Present Studies

In the present two studies, we surveyed middle school students in their math classes about their perceptions of two highly effective math learning strategies—spaced practice and interleaved practice—in the context of hypothetical math assignments. In Study 1, students judged the efficacy and likability of the two strategies. For comparison, we also surveyed their perceptions of more (vs. less) practice, because we suspected that most students would recognize *more practice* as beneficial, if sometimes unlikable. To foreshadow, Study 1 raises concerns about students’ perceptions of interleaved practice, so Study 2 further addressed perceptions of interleaving. Along with perceptions of efficacy and preference, Study 2 examined other perceptions that could help explain why students dislike and fail to recognize the efficacy of interleaved practice, such as perceptions of difficulty, interest, and the time required for interleaved (vs. blocked) practice.

## 2. Study 1

While the efficacy of spacing and interleaving may not always be recognized by students, the strategy of doing practice problems (i.e., better to do more practice than less practice) is intuitive and likely to be viewed as efficacious. Thus, the perceived efficacy of more (vs. less) practice can provide a benchmark for comparison with students’ perceptions of spaced practice and interleaved practice. We suspected that most students would judge more (vs. less) practice to be efficacious, whereas fewer students would recognize the efficacy of spacing and interleaving. We also asked students to judge strategy likability, which is previously unexplored for these math learning strategies. We suspected that the strategies might differ in their likability, though we did not know which strategies would be most or least likable. Importantly, likability adds to the picture of students’ willingness to use each strategy.

### 2.1. Method

#### 2.1.1. Participants

The participants were 174 students in 7th grade math (ages 12–13) at a middle school in a large school district in Florida. The math course was the modal course for grade 7 students at most schools in the district. We sampled eight class sections of the course taught by two teachers (four sections each). The sample size was large enough to give margins of error smaller than 7.5% on estimated proportions of students, at 95% confidence.

#### 2.1.2. Materials

Math practice worksheet. To give students recent experience with interleaved and blocked practice, we created a practice worksheet consisting of 8 math problems (4 blocked and 4 interleaved). Practice problems required students to compute the volume of a cylinder, use the Pythagorean theorem, solve an equation for *x*, compute the volume of a sphere, and identify equivalent mathematical expressions. We also created an answer key showing worked solutions, which we provided to the teachers. Students completed the practice worksheet before taking the survey.

**Figure 1:**
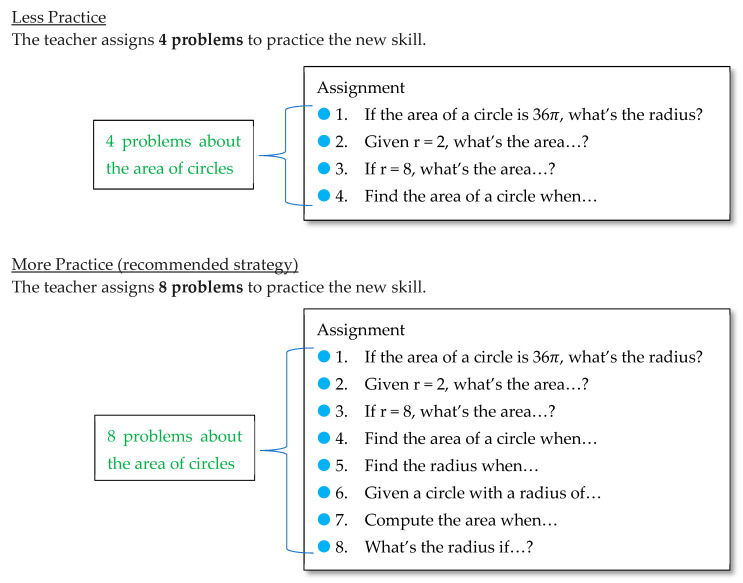
Less Versus More Practice (Study 1). The green words summarize each hypothetical assignment. The blue dots signify that each problem addresses the same new skill (area of circles).

**Figure 2:**
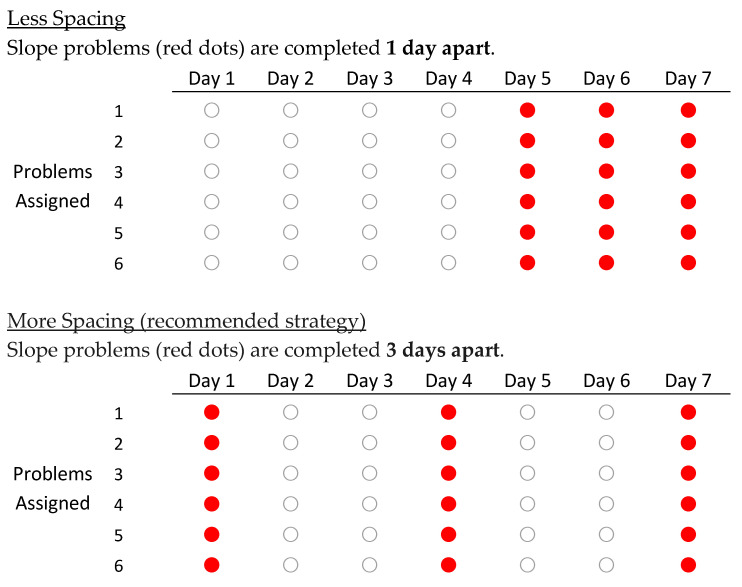
Less Versus More Spacing (Study 1). Red dots represent slope problems and white dots represent various other types of math problems.

**Figure 3:**
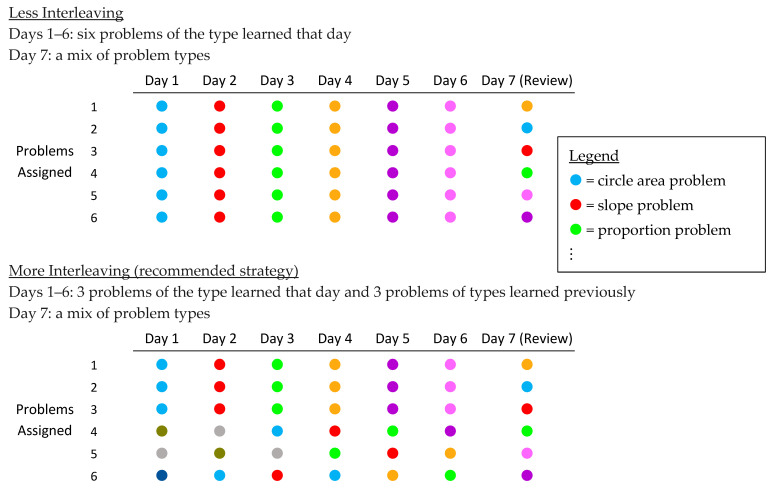
Less Versus More Interleaving (Study 1). Each dot color represents a different type of math problem (see Legend for examples).

Survey booklet. A survey booklet presented three math learning scenarios. The first scenario addressed *more practice*—a strategy we expected students would recognize as efficacious. The second and third scenarios addressed the two strategies of primary interest—*spaced practice* and *interleaved practice*, respectively. Each scenario was presented on its own page and included a short explanation of the scenario followed by two options (labeled “A” and “B”)—a more efficacious option (e.g., more spacing) and a less efficacious option (e.g., less spacing), with the order of the two options counterbalanced (i.e., more efficacious vs. less efficacious option shown first). The options were presented as diagrams like in Figure 1, Figure 2 and Figure 3 (but with “Option A” or “Option B” in place of the underlined labels). In the first scenario, students were told to imagine learning a new skill (e.g., finding the area of a circle) followed by two options (Figure 1): a 4-problem assignment or an 8-problem assignment. In the second scenario, students considered two options for the spacing of slope problems (Figure 2): one day apart or three days apart. In the third scenario, students considered two options for interleaving (Figure 3): 6 problems of the type learned that day or 3 problems learned that day plus 3 problems of previously-learned types (where both options included an interleaved review on Day 7 to equate the recency of each problem type and to increase realism).

For each scenario, students judged (1) perceived efficacy (“Which option would be *more helpful* to prepare you for a test… given a month later?) and (2) likability (“Which option would you *like more*?) by selecting among 5 responses that corresponded to the chosen option (A or B) and strength of the choice (much more, slightly more, equal). The order of the two judgments (i.e., efficacy, likability) was counterbalanced. The complete survey with instructions and administration details, as well as the data for both studies, can be found at https://osf.io/rwbq4/ (accessed on 3 June 2025).

#### 2.1.3. Procedure

The study occurred during a class period without the researchers present. The teacher distributed the math practice worksheet to each student in the participating class. Students were instructed to try solving the practice problems and were permitted to work together and seek help if needed. When most students were finished or no longer making progress on the problems, the teacher then distributed a survey booklet to each student and provided an oral explanation (prepared by the researchers) that guided students through each page. The oral explanation for each scenario explained to students what the various dots represented (e.g., in the spacing scenario, red dots represented slope problems and white dots represented various other types of math problems; in the interleaving scenario, example topics were given for different dot colors). After students completed the survey, the teacher collected the booklets. Finally, the teacher presented the solutions to the math problems on the practice worksheet. The entire procedure (i.e., practice worksheet, survey, and problem solutions) required approximately 40 min and was completed within one class period. The survey booklets were returned to the researchers, but the practice worksheets were not.

### 2.2. Results and Discussion

**Figure 4:**
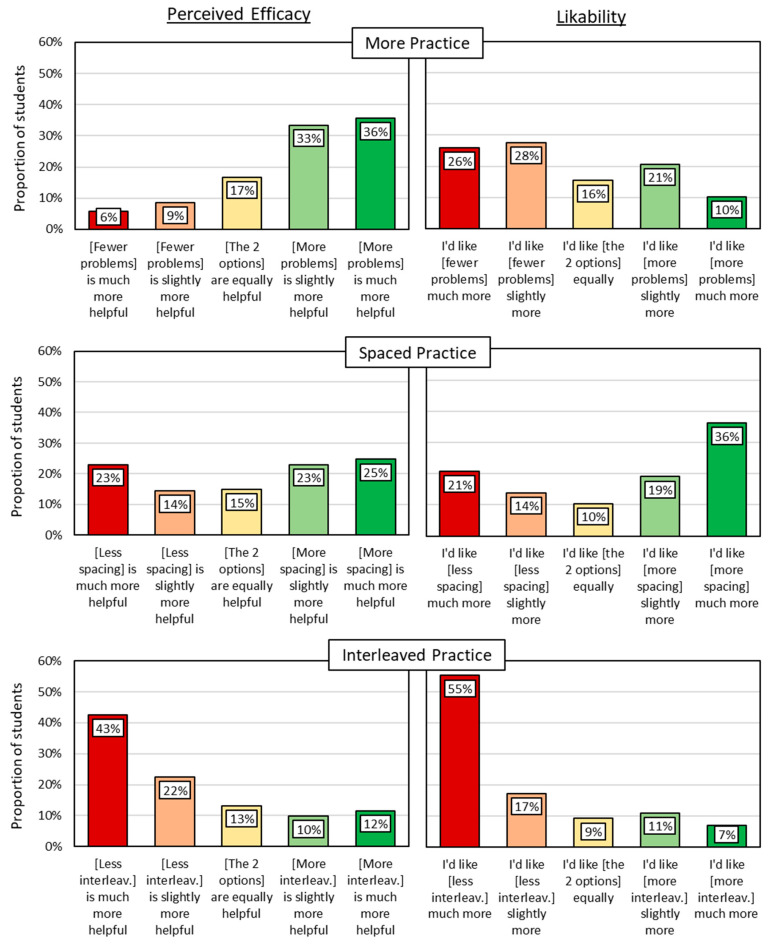
Perceived Efficacy and Likability of Math Learning Strategies (Study 1).

Students’ perceptions of the efficacy of each strategy are shown in the left column of Figure 4. For the strategy of doing more practice (vs. less practice), 69% of students said the option with more problems was slightly or much more effective than the alternative. In other words, most (but not all) students recognized the efficacy of doing practice problems—approximately as expected. For the strategy of spacing, however, students’ perceptions of efficacy were more varied, with responses relatively evenly distributed across the five response choices, indicating that students’ insight into the efficacy of spacing was limited at best. Chi Square goodness-of-fit tests were used to determine whether the response pattern for each survey question differed from random responding, and only the question about spacing efficacy was indistinguishable from random (see OSF for details), which could reflect students’ differing opinions or uncertainty about spacing efficacy. Finally, for the strategy of interleaving, only 22% said the option with more interleaving was slightly or much more effective, whereas 65% said the option with little interleaving (i.e., mostly blocked) was more effective, contrary to actual efficacy. Thus, most students had erroneous perceptions about the efficacy of interleaving.

Students’ perceptions of likability of each strategy are shown in the right column of Figure 4. Regarding the quantity of practice problems, more than half of students preferred the less efficacious strategy of doing fewer (rather than more) problems. Regarding spacing, however, more than half of students preferred the more efficacious strategy of more spacing (rather than less spacing). Finally, regarding interleaving, students overwhelmingly (72%) preferred the less efficacious strategy of mostly blocked practice (rather than interleaved practice).

In summary, the middle school students in Study 1 were mostly aware that doing practice problems helps their learning, even if they do not always like it. Concerning spaced practice, many students found it likable, yet they had no consensus about its efficacy. This result signals an opportunity to educate students about the efficacy of spacing which, given its likability, may face little resistance from students. The results for interleaving, however, were more troubling. Students often erroneously believed that learning was benefitted by less (rather than more) interleaving, and this misperception about interleaving efficacy was paired with low likability. These results paint a grim picture regarding students’ inclination for interleaved practice. In Study 2, we surveyed additional perceptions that could help explain the low perceived efficacy and low likability of interleaved practice. For example, students might perceive interleaved practice to be more difficult or time-consuming than blocked practice, and such perceptions might be interpreted by students as evidence of low efficacy (E. L. Bjork & Bjork, 2011; Kirk-Johnson et al., 2019) and contribute to low likability. As in Study 1, we again relied on diagrams to help students understand the strategy in question, avoiding words like “interleaving” that may be confusing or unfamiliar to them. Unlike Study 1, however, Study 2 used an alternative diagram offering a simpler depiction of interleaved and blocked practice worksheets.

## 3. Study 2

**Figure 5:**
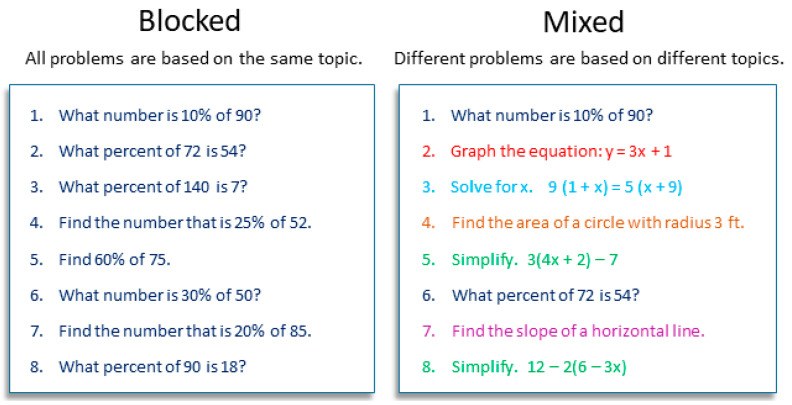
Example of Blocked versus Interleaved (Mixed) Practice (Study 2).

Students’ negative perceptions of interleaved practice—as unlikable and inefficacious—are concerning and merit additional investigation, so in Study 2 we further explored perceptions of interleaved practice. We again surveyed middle school math students, but we used a different illustration to depict interleaved (“mixed”) practice and its alternative, blocked practice (see Figure 5). Using this illustration, we queried students about the efficacy of the two alternatives, students’ preference^2^ between them, the time required to complete them, how difficult they are, and how interesting they are.

### 3.1. Method

#### 3.1.1. Participants

Participants were 233 students in 7th grade math (ages 12–13) at a middle school in a large school district in Florida. The school was the same as in Study 1 but during a different school year, and the two studies did not include any of the same participants. The math course was the modal course for grade 7 students at most schools in the district. We sampled twelve class sections of the course taught by four teachers (two to four sections each). The sample size was large enough to give margins of error smaller than 6.5% on estimated proportions of students, at 95% confidence.

The survey reported here was part of a larger, classroom-based, full-year study that ended unexpectedly, before we could administer the classroom-based final test, when the coronavirus pandemic forced schools to close abruptly and move all instruction online for the remainder of the school year. In its place, the participating teachers agreed to an online survey reported here. The original study involved an in-person, within-subjects manipulation of math practice in the classroom. It was not expected to affect survey responses and occurred 8 weeks before the survey was administered.

Of the 260 students in the original study, 234 completed the online survey (90%). One participant was excluded from analysis for spending less than 10 s on the entire survey, which we believe was too little time to examine the diagram, read the survey questions, and give meaningful responses. Our final sample consisted of 233 students.

#### 3.1.2. Materials and Procedure

**Figure 6:**
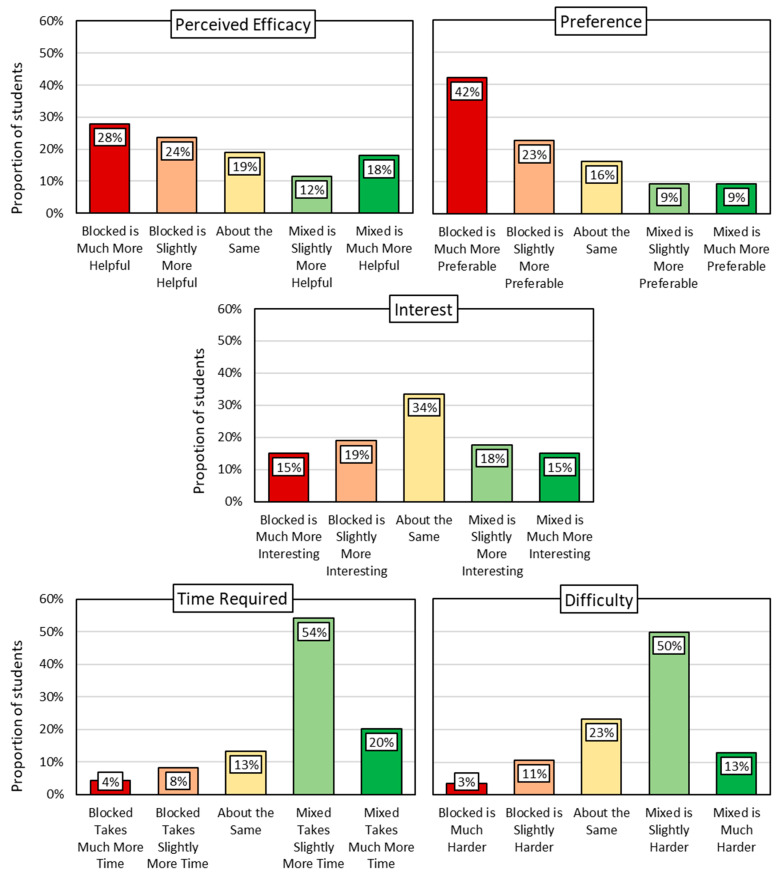
Blocked vs. Interleaved Practice (Study 2).

A survey link was given to teachers who distributed it to the students in their participating classes. Students could complete the survey at any time during the week it was assigned. The survey took less than 5 min and was followed by an online math task unrelated to the survey. The survey presented students with a diagram of hypothetical interleaved and blocked assignments (see Figure 5), along with a brief description of each. With the diagram still visible, students were asked to make five comparisons of the two assignments: time (“Which kind of assignment takes you more time to finish?”), difficulty (“…is harder for you?”), interest (“…is more interesting?”), efficacy (“…helps you score higher on [standardized test^3^]?”), and preference (“…do you prefer?”). (Response options for each question are visible in Figure 6, and a copy of the survey is available at the OSF link provided above.) As a reward for participation, participants received a small gift bag of school supplies at the end of the school year.

### 3.2. Results and Discussion

Students’ survey responses are shown in Figure 6. Like in Study 1, most students in Study 2 failed to recognize the greater efficacy of interleaved practice over blocked practice: only 30% of students said that interleaved practice is slightly or much more effective than blocked, whereas 52% said the opposite. Students also reported a clear preference for blocked practice (65% preferring blocked vs. 18% preferring interleaved). Regarding interest, assignment type did not have a consistent impact: 34% said blocked assignments are more interesting, 33% said interleaved assignments are more interesting, and 34% said they are about the same. Most students agreed, however, that interleaved assignments require more time (74%) and are more difficult (63%).

We also examined the associations among the five perceptions surveyed (Table 1). Students who believed that interleaved assignments take more time (than blocked practice) also generally believed that interleaved assignments are more difficult (*r* = 0.66). Interestingly, perceiving interleaved practice as more time-consuming (less efficient) and more difficult was more strongly associated with low preference than with low perceived efficacy. In other words, although difficulty may indeed be interpreted by students as signaling low strategy efficacy, difficulty may diminish preference even more. Students who more strongly preferred interleaved practice (over blocked) viewed it as less difficult (*r* = −0.61) and less time consuming (*r* = −0.57) but more interesting (*r* = 0.51) and more effective (*r* = 0.48). Altogether, these results suggest that to encourage the use of interleaved practice, researchers and educators must not only convince students of its efficacy but also find ways to overcome negativity pertaining to difficulty, inefficiency, and unlikability.

## 4. General Discussion

In the studies presented here, we investigated middle school students’ perceptions of spaced and interleaved practice—two highly effective math learning strategies. In Study 1, spaced practice was often judged likable, but nearly half of students failed to recognize its efficacy, whereas interleaved practice was judged both unlikable and inefficacious by most students. In Study 2, further exploration of students’ perceptions of interleaved practice pointed to possible reasons that most students dislike interleaving and fail to perceive its efficacy—including perceptions that interleaved assignments are time-consuming and difficult. Understanding students’ perceptions of learning strategies is important because these perceptions can influence whether the strategies get used (Zepeda et al., 2020). The present findings highlight the need to consider not only students’ metacognitive knowledge of strategy efficacy but also strategy likability.

With respect to students’ perceptions of strategy efficacy, the present work suggests that inadequate metacognitive knowledge may be a barrier for both spaced and interleaved practice, albeit to different degrees. When students compared more spaced versus less spaced practice, they lacked a consensus regarding efficacy, suggesting that, for at least half the students, their strategy knowledge needs improvement. The strategy knowledge of these 7th graders might, at first glance, seem deficient compared to college students who recognize the efficacy of *spaced over massed* practice (e.g., Susser & McCabe, 2013), but recognizing the efficacy of *more spacing over less spacing* is difficult for college students also (e.g., Hartwig et al., 2022). With interleaved practice, perceptions of efficacy were even more concerning: most students (65%) erroneously believed that less interleaved (mostly blocked) practice was superior to more interleaved practice (Study 1), and even a clear-cut comparison of fully blocked vs. fully interleaved assignments (Study 2) found that more than half of the students perceived blocked to be superior. This is consistent with prior work showing that students commonly draw the wrong conclusion about interleaved vs. blocked efficacy (e.g., Kornell & Bjork, 2008) and that dissuading them from this misperception is difficult (Yan et al., 2016). How to best remediate students’ erroneous metacognitive beliefs about spacing and interleaving should be studied in future work to provide a basis for successful intervention (see McDaniel & Einstein, 2020, for further discussion).

The likability of a strategy also affects its use (Obergriesser & Stoeger, 2020), and in the present study, likability was shown to differ considerably by strategy. Spacing was judged favorably, with more than half of students in Study 1 reporting greater liking for practice that is more spaced rather than less spaced. This suggests that likability is not a large barrier for spaced practice. In contrast, the likability of interleaved practice does appear to be a substantial hurdle, with 72% of students liking practice that is less interleaved (vs. more interleaved; Study 1) and 65% preferring blocked assignments (vs. interleaved assignments; Study 2). Finding ways to increase the likability of interleaved practice is an important future direction to encourage its use.

We also explored other potential barriers for interleaved practice—including perceptions that the strategy is more difficult, more time-consuming, or less interesting than blocked practice. Indeed, interleaved practice was judged by more than 60% of students to be more difficult and more time-consuming than blocked practice—and, importantly, students are correct in these perceptions. Interleaved assignments demand switching between different problem types and identifying an appropriate approach for each one, which is more time-consuming and effortful compared to a blocked assignment that repeats one problem type over and over. These inherent attributes of interleaved practice (difficult, time-consuming) may discourage its use, especially if these attributes are interpreted as signs of low efficacy or generate dislike. Consistent with prior work, we found that higher perceived difficulty was associated with lower perceived efficacy (*r* = −0.28; see Table 1), highlighting that high effort is commonly misinterpreted as poor learning (Kirk-Johnson et al., 2019). Perceived difficulty was also negatively associated with low preference for interleaved (vs. blocked) practice (*r* = −0.61), suggesting that the difficulty of interleaved practice contributes to its low likability. Getting students to embrace the difficulty that is often inherent in effective learning strategies will be important not only for improving students’ knowledge of strategy efficacy but also (and perhaps more) important for increasing overall strategy likability and preference.

### 4.1. Limitations

Our survey focused on 7th graders’ perceptions of math learning strategies, so it is reasonable to ask whether our findings would generalize to students of other ages or to other subject areas. Students’ strategy perceptions could change across grade levels as students acquire new learning experiences that shape their beliefs and expectations (Schneider, 2008). That said, even college students have difficulty recognizing the efficacy of spaced and interleaved math practice (Hartwig et al., 2022), so experience with school in general or math learning specifically might not, by itself, markedly change these strategy beliefs. Whether the same is true for strategy likability is unclear, though if likability is shaped by the perceived difficulty or efficiency of a strategy, we might predict continuity for likability as well. With respect to other subject areas, an interesting question is whether norms of practice within different fields of study might create different expectations and strategy beliefs among students within those fields. Notably, in the field of math (and other computational fields, such as chemistry or physics) students are commonly assigned practice problems, which serve as small units of practice that can be easily spaced across time or interleaved within assignments, providing many opportunities to encounter these learning strategies. Despite likely exposure to spaced and interleaved practice in the context of math learning, students still tend to undervalue these strategies. It may be useful for future work to verify whether spacing and interleaving are similarly undervalued by students in other fields, since these strategies are useful for other subject areas also (e.g., Carpenter et al., 2012; Eglington & Kang, 2017; Pan et al., 2019).

The present studies did not test whether the math strategy perceptions we surveyed did indeed affect actual use of spaced and interleaved practice, so we cannot say to what extent these perceptions mapped onto behavior. Rather, we rely on prior theorizing, models of self-regulated learning, and related work (e.g., McDaniel & Einstein, 2020; Obergriesser & Stoeger, 2020; Panadero, 2017; Zepeda et al., 2020) to underpin the assertion that students’ strategy perceptions influence their strategy choices and that a strategy viewed as ineffective or unlikable will be used less often. Plausibly, students’ strategy perceptions are less consequential when external forces (such as teachers’ lesson plans or textbook assignments) dictate the type of practice that occurs. However, in many learning situations, students are decision-makers about the type of study they choose or the mental activities they engage—and their learning-related perceptions may become increasingly important as they get older and their learning activities become more self-regulated. Even when teachers do heavily shape the practice that occurs, teachers themselves can be affected by students’ perspectives and attitudes toward specific learning strategies, opting to encourage those strategies their students favor rather than protest. An interesting direction for further work may include teachers’ perceptions of learning strategies (Halamish, 2018; Morehead et al., 2016; Rohrer et al., 2020b) and how teachers’ choices are affected by their students’ perceptions.

Also worth considering is whether participants could be justified in perceiving greater efficacy for strategy options we regarded as less efficacious. Though evidence from prior research is strong in support of spaced and interleaved practice, we did not verify the efficacy of the options shown in our scenarios. The first scenario in Study 1 may not always favor more (rather than less) practice, as prior work on overlearning has shown diminishing returns of consecutive repetitions of the same problem type within a single session (e.g., Rohrer & Taylor, 2006). Thus, the small proportion of students (15%) who perceived greater efficacy for fewer problems might have prioritized efficiency, and those who viewed the options as equally helpful (17%) may have correctly seen no additional benefit from extra problems. That said, the problems in that scenario (Figure 1) varied in form (sometimes requiring solving for area, other times solving for radius), so overlearning may be less relevant. With the interleaved scenario (Figure 3), participants could justifiably question the efficacy of the interleaved option if they noticed that topics taught later (e.g., on day 6) received less practice compared to earlier topics and compared to the same topics in the blocked option. This difference, typical of interleaving in classrooms, occurs because the first assignments must interleave with previously-taught topics (from a previous exam or previous school year) rather than with topics not-yet-taught, and later topics have fewer days available for interleaved practice before an exam (though they may be interleaved in future assignments beyond that exam). This non-equivalence between blocked and interleaved practice could have contributed to participants’ perceptions of lower efficacy for interleaving, and future work might design scenarios with better equivalence. Regardless, effect sizes for interleaved (vs. blocked) practice are typically large and unlikely to be reversed by a small reduction of practice for later topics. Finally, misjudgments of efficacy could also result from misunderstanding the diagrams. For instance, when shown blocked and interleaved sample worksheets (Figure 5), did participants think that interleaving implied less practice or less instruction for each problem type? Future work may aim to clarify such details. In sum, we cannot say with certainty that participants’ misperceptions of efficacy were entirely unjustified. However, our use of different diagrams across two studies offers some assurance that misperceptions were robust and not due to a single detail. As discussed above, our findings align with prior studies, using other methods, that also conclude that students underestimate the efficacy of interleaved practice.

### 4.2. Implications

Because students’ perceptions of learning strategies can affect whether those strategies are used, it is important to understand how the most effective strategies are perceived by students. Interleaved practice, a highly effective strategy for math learning, may meet resistance because students dislike it and fail to recognize its efficacy. To promote interleaved practice, the challenge is, at minimum, twofold: convince learners of its efficacy and increase its palatability. To convince learners of its efficacy, approaches might include study strategy training or learning demonstrations, as well as explaining to students the value of the effort involved in interleaved practice. Approaches to improve the palatability of interleaved practice might include scaffolding, teamwork, or gamification (for more suggestions, see Zepeda et al., 2020).

In contrast, spaced practice may meet less resistance because students find it palatable, even if they are not always aware of the superior efficacy of more spaced (vs. less spaced) practice. To promote spacing, training and demonstrations may help teach its efficacy; but it is also possible that other barriers not examined here may pose a challenge for spacing. For example, when studying on their own, students may find it difficult to summon the discipline required to implement a continued schedule of spaced practice across time. Effective interventions will be those that help learners overcome the specific barriers that hinder the use of effective learning strategies. Importantly, as we show here, effective learning strategies do not all have the same barriers, and interventions should be tailored accordingly.

## Figures and Tables

**Table 1:** Correlations Among Perceptions of Interleaved Practice (Study 2).

Perceptions	1	2	3	4	5
1. Time Required	–				
2. Difficulty	0.66 *	–			
3. Interest	−0.30 *	−0.41 *	–		
4. Efficacy	−0.30 *	−0.28 *	0.33 *	–	
5. Preference	−0.57 *	−0.61 *	0.51 *	0.48 *	–

* *p* < 0.01.

## Data Availability

The original data presented in these studies are openly available at https://osf.io/rwbq4/ (accessed on 3 June 2025).

## Notes

1 We note that the superiority of interleaved over blocked practice does not hold in all contexts. For example, blocking can outperform interleaving when learners must abstract and apply rules that define category membership, especially if within-category similarity is low (for further discussion, see Little & Nepangue, 2025; Noh et al., 2016). However, in classrooms, students are usually explicitly taught the rules that define to-be-learned concepts or categories rather than having to abstract them. Thus, for math learning in schools, which is the focus of this paper, we would expect the typical benefit of interleaved over blocked practice.

2 Judgments of preference (Study 2) were considered comparable to judgments of liking (Study 1), though we acknowledge that preference might be shaped by other factors in addition to liking, including familiarity or other justifications for favorability.

3 We inserted the name of a state standardized test that was familiar to the students in this study.
